# Posterior interosseous nerve palsy caused by synovial osteochondromatosis of the elbow analyzed by three-dimensional reconstruction: a case report

**DOI:** 10.1186/s13256-018-1865-y

**Published:** 2018-11-19

**Authors:** Koichi Yano, Yasunori Kaneshiro, Kosuke Sasaki, Hideki Sakanaka

**Affiliations:** 1 Department of Orthopaedic Surgery, Seikeikai Hospital, 1-1-1 Minamiyasuicho, Sakai-ku, Sakai City, Osaka 590-0064 Japan; 2 Department of Orthopaedic Surgery, Ekisaikai Hospital, 2-1-10 Honda, Nishi-ku, Osaka City, Osaka 550-0022 Japan

**Keywords:** Synovial osteochondromatosis, Posterior interosseous nerve palsy, Elbow, Three-dimensional reconstruction

## Abstract

**Background:**

Synovial osteochondromatosis, a benign tumor consisting of cartilage and bone, generally presents as multiple osteochondral or chondral nodules. Peripheral nerve palsy caused by synovial osteochondromatosis is rare. Three-dimensional reconstruction based on magnetic resonance imaging shows the specific shape and location of the tumor and its relation to the nerve.

**Case presentation:**

We describe a case of posterior interosseous nerve palsy caused by synovial osteochondromatosis of the elbow in a 66-year-old Japanese man. A three-dimensional reconstructed image based on magnetic resonance imaging was used to determine the location and shape of the giant tumor, which was composed of bone and cartilage. After surgical resection of the giant tumor and neurolysis of the posterior interosseous nerve, he fully recovered from nerve palsy 9 months postoperatively. There was no recurrence of the lesion 1 year postoperatively.

**Conclusion:**

Synovial osteochondromatosis that causes posterior interosseous nerve palsy has a characteristic morphology and location, that is, a giant tumor located anterior to the humeroradial joint, as revealed by three-dimensional magnetic resonance image reconstruction.

## Background

Synovial osteochondromatosis is a benign tumor consisting of cartilage and bone that arises from the synovium of the joint, bursae, and tendon sheath. In general, it presents as multiple osteochondral or chondral nodules attached to the synovium [1, 2]. One joint is affected, and the most commonly affected joint is the knee, followed by the hip and elbow joints. Loose bodies in the joint can cause pain, swelling, a palpable mass, locking or damage to the articular surface, and limitations in motion [1, 2]. A lesion of the elbow rarely causes peripheral nerve palsy, and the three-dimensional shape of the tumor has not been understood clearly [3–7].

We describe a case of posterior interosseous nerve (PIN) palsy caused by synovial osteochondromatosis of the elbow, and we discuss the usefulness of three-dimensional reconstruction based on magnetic resonance imaging (MRI) for clear visualization of the specific shape and location of the tumor and its relation to the PIN.

## Case presentation

A right-handed 66-year-old Japanese man experienced right elbow pain and was unable to extend his right thumb and fingers for 1 month. He did not have associated history of trauma to his elbow or any remarkable medical history. A physical examination showed swelling of his right elbow and a palpable mass on the anterior aspect of his right elbow. Grip strengths of his right and left hands, as measured with a Jamar digital dynamometer (Takei Scientific Instruments Co., Ltd., Niigata, Japan), were 30.4 and 35.0 kg, respectively. The respective ranges of motion for his right and left extremities, as measured with a standard goniometer, were as follows: elbow flexion, 115° and 145°; elbow extension, − 15° and 0°; forearm pronation, 30° and 70°; forearm supination, 80° and 90°. The muscle strengths of his right upper extremity, as evaluated using the British Medical Research Council scale, were the following: triceps, 5; wrist extensor, 5; extensor pollicis longus, 3; extensor digitorum communis and extensor indicis proprius (EIP), 2. There was no sensory loss.

**Fig. 1:**
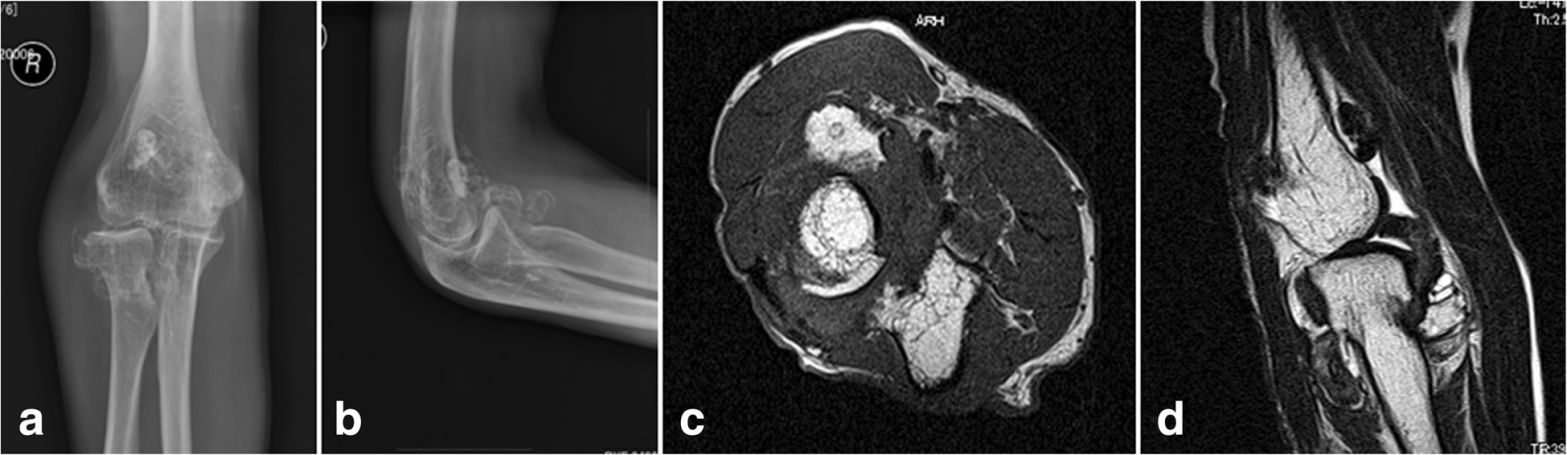
Preoperative plain radiographs and plain magnetic resonance images of the right elbow. **a** Anteroposterior view and **b** lateral view of plain radiographs. Osteoarthritic change is visible with ectopic calcifications and ossicles anteriorly, posteriorly, and laterally in the elbow. **c** Axial view and **d** sagittal view of the plain magnetic resonance images. A mass lesion is visible around the radial head and neck, anterior to the humerus, and in the olecranon fossa, with heterogeneous intensity on T1-weighted and T2-weighted images

Plain radiographs of his right elbow showed osteoarthritic change with calcifications and ossicles anteriorly, posteriorly, and laterally (Fig. 1a, b). Plain computed tomography (CT) and three-dimensional CT scans (Activion 16; Toshiba Medical Systems Corp., Tokyo, Japan) showed clustered calcifications around the radial neck, coronoid fossa, radial fossa, and olecranon fossa. Plain magnetic resonance imaging (MRI) scans (EXCELART Vantage 1.5 Tesla, version 9.51; Toshiba Medical Systems Corp.) showed mass lesions around the radial neck, medial epicondyle, olecranon fossa, and coronoid fossa, with heterogeneous intensity on T1-weighted and T2-weighted images (Fig. 1c, d). A nerve conduction study (NCS) for his radial nerve was performed. For the motor NCS, which was recorded at the EIP, surface electrodes for stimulation were set at 8 cm proximal to the EIP, 5 cm proximal to the elbow crease, and posterior to the insertion of the deltoid. For the antidromic sensory NCS, it was recorded at the middle between the first and second metacarpal bones; surface electrode for stimulation was set at 14 cm proximal to the recording position. The respective right and left motor nerve conduction velocities were 70.2 and 57.7 m/s from the upper arm to the elbow and 29.9 and 66.1 m/s from the elbow to the forearm. The results of sensory nerve conduction velocities were 62.5 and 57.9 m/s, respectively.

**Fig. 2:**
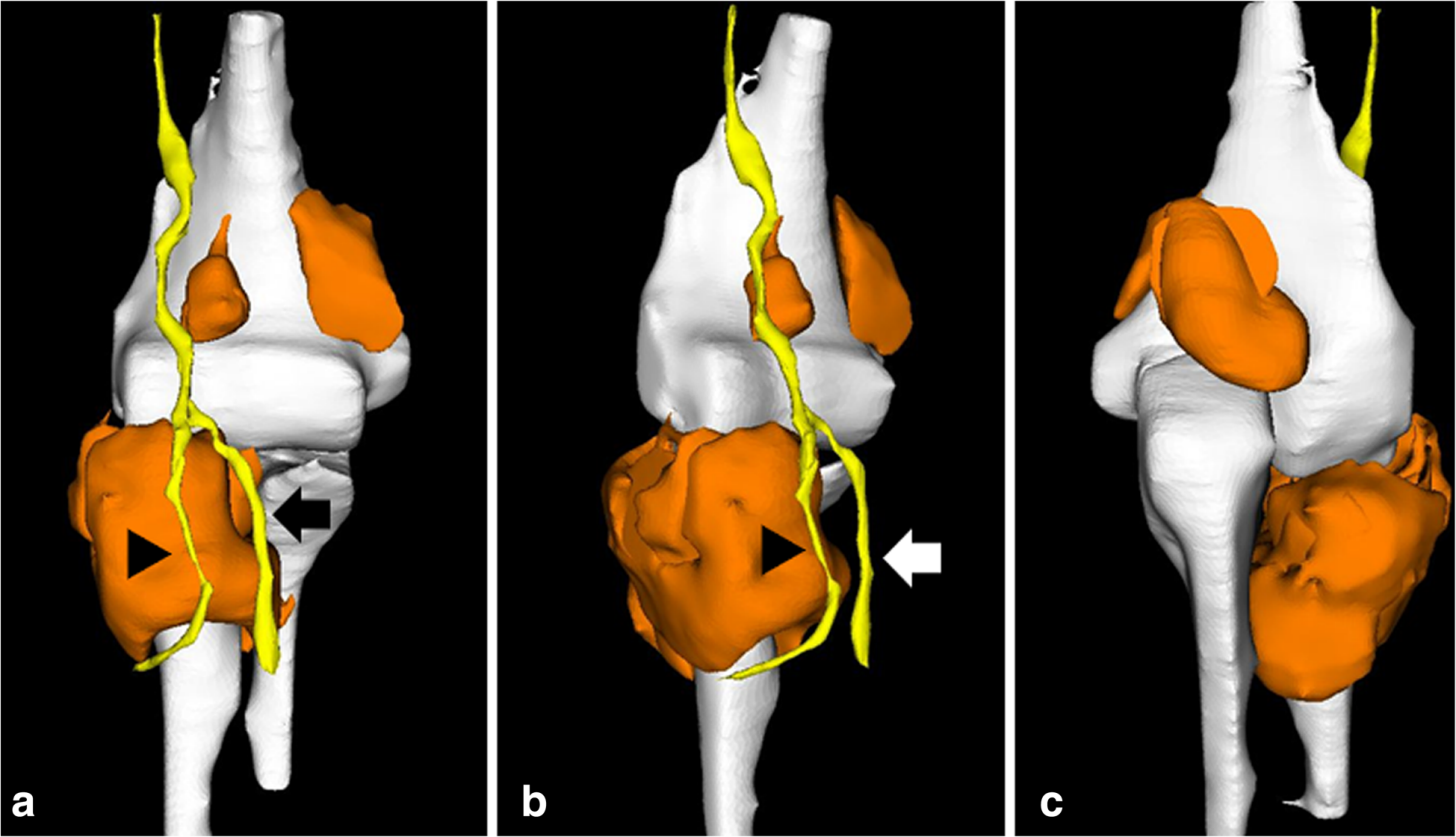
Three-dimensional images based on magnetic resonance imaging of the right elbow. **a** Anterior image. **b** Oblique image. **c** Posterior image. The tumor is orange; the bone, humerus, radius, and ulna are white; and the nerve is yellow. The running course of the posterior interosseous nerve (*arrow head*) is changed at the distal corner of the tumor in contrast to the superficial branch of radial nerve (*arrow*)

The quality of the three-dimensional CT images was poor for detecting the tumor so we created three-dimensional reconstructed images of the tumor based on MRI to visualize the shape and location of the tumor and its relation to the radial nerve. Digital imaging and communications in medicine data obtained from plain magnetic resonance (MR) images were transferred to Mimics computer-aided design (CAD) software (Materialise Japan, Yokohama, Japan). The bone region was segmented semi-automatically with an intensity threshold segmentation technique, and the tumor and nerve (radial nerve, PIN, and superficial branch of radial nerve) intensity was contoured manually using the CAD software; then, three-dimensional reconstruction was performed (Fig. 2a–c). The three-dimensional images clearly showed the tumor location and morphology; we found a giant tumor around the radial head and neck and a large mass in the radial fossa, olecranon fossa, and medial to the coronoid fossa. Moreover, the running course of the PIN was extremely changed at the distal corner of the tumor in contrast to the superficial branch of the radial nerve.

We diagnosed our patient as having incomplete PIN palsy caused by synovial osteochondromatosis. We decided to resect the tumor from the anterolateral part and radial fossa and perform neurolysis of the PIN because our patient had no impairment in daily life, for example, limitations in range of motion were compensated by shoulder joint motion.

**Fig. 3:**
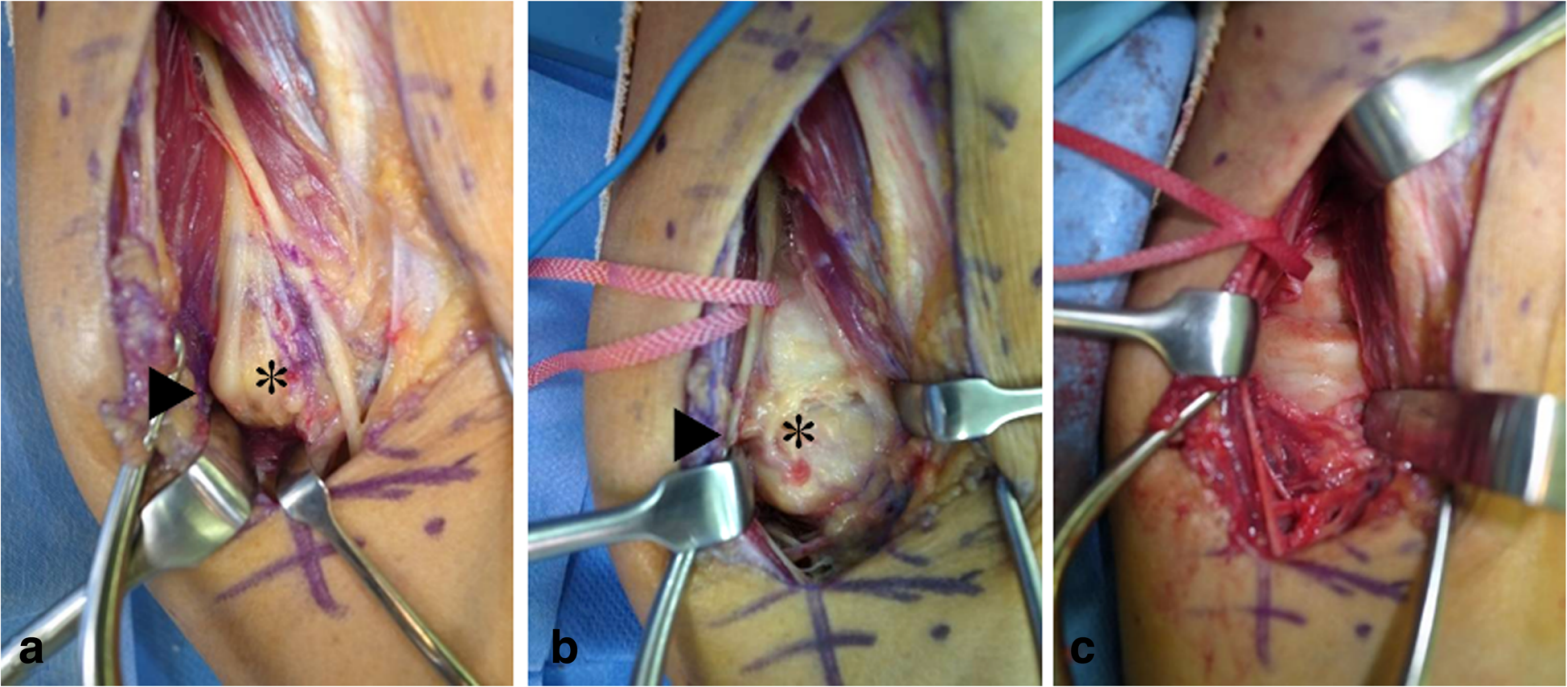
Intraoperative photographs. **a** The posterior interosseous nerve (posterior interosseous nerve, *arrowhead*) is compressed between the arcade of Frohse and the tumor (*asterisk*), and the posterior interosseous nerve is kinked at the corner of the tumor. **b** A white, cartilaginous tumor (*asterisk*) is shown after neurolysis of the posterior interosseous nerve (*arrowhead*) and incision of the capsule was performed. **c** The radial head and annular ligament are shown after tumor resection

Surgical treatment was performed via the anterolateral (Henry) approach using an air tourniquet, with our patient under general anesthesia. The three-dimensional reconstruction images were used as a basis for the surgical exposure, and the PIN was compressed between the arcade of Frohse and the tumor, which was under the PIN (Fig. 3a). The PIN was kinked, especially at the corner of the tumor, as shown by the three-dimensional reconstruction image. After the incision of the arcade of Frohse was made, neurolysis of the PIN was performed. The tumor was covered by the joint capsule (Fig. 3b). When the joint capsule was incised, a white, cartilaginous tumor was found. The tumor, including the synovium, was resected (Fig. 3c). Results of a histological analysis showed synovial osteochondromatosis without malignancy. No major postoperative complications occurred. Our patient fully recovered from the PIN palsy 9 months postoperatively. One year postoperatively, his grip strengths were 36.7 and 33.7 kg for his right and left hands, respectively. The ranges of motion of his right extremity were as follows: elbow flexion, 125°; elbow extension, − 15°; forearm pronation, 65°; forearm supination, 90°. No recurrence of the lesion occurred 1 year postoperatively.

## Discussion

Peripheral nerve palsy due to synovial osteochondromatosis of the elbow joint is rare, and there are some case reports of palsy of the PIN [3–5, 7], ulnar nerve [6, 8–13], and median nerve [14].

Regarding PIN palsy caused by synovial osteochondromatosis of the elbow joint, only four cases have been reported in the English literature [3–5, 7] (Table 1). The average age of the patients was 50 years (range, 46–56 years); three of the patients are women and one is a man. The duration of the preoperative symptoms ranged from 10 days to 4 months. All patients were treated surgically, and the operations consisted of neurolysis of the PIN and tumor excision. The tumors existed anterior to the humeroradial joint, and the PIN was compressed between the arcade of Frohse and the tumor, as observed in our case. The follow-up period ranged from 9 months to 3.5 years. All patients fully recovered from PIN palsy. Overall, the clinical outcomes were good, similar to those in our case.

**Table 1:** Demographic data of previously reported cases of posterior interosseous nerve palsy caused by synovial osteochondromatosis of the elbow

Case	Age	Sex	Side	Duration of the preoperative symptom	Elbow pain	Follow-up period	Outcome of nerve palsy
1 [3]	48	F	R	10 days	+	6 months	Full recovery
2 [4]	49	M	R	ND	NA	1 year	Full recovery
3 [5]	56	F	R	4 months	+	9 months	Full recovery
4 [7]	46	F	L	1 month	NA	2.5 years	Full recovery
Our case	66	M	R	1 month	+	1 year	Full recovery

*F* female, *M* male, *NA* not applicable, *ND* no data, *L* left, *R* right

Although multiple loose bodies are generally found in symptomatic patients with synovial osteochondromatosis, the morphological pattern of a tumor causing PIN palsy is specific. A giant solitary tumor was found in two patients (similar to that observed in our patient); large, loose bodies were found in one patient; and the morphological pattern was not described in one patient. Milgram described the following three phases of synovial osteochondromatosis, based on a histopathological study: phase 1, an active intrasynovial disease with no loose bodies; phase 2, the transitional stage involving active intrasynovial disease and a loose body; phase 3, multiple osteochondral loose bodies without active synovial disease [1]. Subsequently, Edeiken *et al.* added phase 4, which was defined as a large intra-articular or extra-articular calcified cartilaginous mass that can be formed by the fusion of multiple synovial chondromas or by the growth of a solitary synovial chondroma [15]. In four of the five cases of PIN palsy, the synovial osteochondromatosis could be categorized as phase 4; thus, PIN palsy may be caused by a giant tumor anterior to the humeroradial joint.

Morphological assessment of the bone tumor can be obtained by three-dimensional reconstruction based on CT; however, a cartilaginous tumor cannot be visualized using CT. When the tumor is mainly composed of cartilage, a three-dimensional image based on MRI provides better visibility compared with that based on CT and demonstrates the relation between the peripheral nerve and the tumor in a case with peripheral nerve palsy. Recently developed computer technology enables surgeons to convert two-dimensional images into three-dimensional images using software [16]. This application uses MR images and has been used for assessing the preoperative morphology of a pituitary macroadenoma and musculoskeletal tumor [17, 18]. To the best of our knowledge, there has been no report concerning three-dimensional reconstruction of a chondroma, osteochondroma, and synovial osteochondromatosis or chondromatosis. The anatomical relationship between the PIN and the tumor was especially important for determining the pathology of our case. The three-dimensional image based on MRI was useful for detecting the location and morphology of the tumor and its relation to the nerve, showing the clinical issue to our patient, and sharing the information among surgeons.

The case report has several limitations. First, we could not compare the postoperative condition of the PIN with its preoperative status because we did not perform postoperative MRI. Second, we did not perform magnetic resonance neurography. A recent clinical study reported that magnetic resonance neurography was useful for assessing peripheral nerves [19]. Careful follow-up of the remaining tumors located medial and posterior to the elbow is necessary.

## Conclusions

PIN palsy caused by synovial osteochondromatosis of the elbow is a rare condition. After surgical resection of the giant tumor and neurolysis of the PIN, our patient fully recovered from nerve palsy 9 months postoperatively. A three-dimensional reconstructed image based on MRI shows that synovial osteochondromatosis that causes PIN palsy has a characteristic morphology and location, that is, a giant tumor located anterior to the humeroradial joint.

## Abbreviations

CAD: Computer-aided design
CT: Computed tomography
EIP: Extensor indicis proprius
MR: Magnetic resonance
MRI: Magnetic resonance imaging
NCS: Nerve conduction study
PIN: Posterior interosseous nerve
